# A subpopulation of cortical neurons altered by mutations in the autism risk gene *DDX3X*

**DOI:** 10.1242/bio.061854

**Published:** 2025-01-29

**Authors:** Michael A. Flores, Marta Garcia-Forn, Alexa von Mueffling, Praise Ola, Yeaji Park, Andrea Boitnott, Silvia De Rubeis

**Affiliations:** ^1^ Seaver Autism Center for Research and Treatment, Icahn School of Medicine at Mount Sinai, New York, NY 10029, USA; ^2^ Department of Psychiatry, Icahn School of Medicine at Mount Sinai, New York, NY 10029, USA; ^3^ The Mindich Child Health and Development Institute, Icahn School of Medicine at Mount Sinai, New York, NY 10029, USA; ^4^ Friedman Brain Institute, Icahn School of Medicine at Mount Sinai, New York, NY 10029, USA; ^5^ The Alper Center for Neural Development and Regeneration, Icahn School of Medicine at Mount Sinai, New York, NY 10029, USA

**Keywords:** Cortical development, DDX3X syndrome, Intellectual disability, Neurodevelopment, Subcerebral projections

## Abstract

Cell fate decisions during cortical development sculpt the identity of long-range connections that subserve complex behaviors. These decisions are largely dictated by mutually exclusive transcription factors, including CTIP2/*Bcl11b* for subcerebral projection neurons and BRN1/*Pou3f3* for intra-telencephalic projection neurons. We have recently reported that the balance of cortical CTIP2-expressing neurons is altered in a mouse model of DDX3X syndrome, a female-biased neurodevelopmental disorder associated with intellectual disability, autism spectrum disorder, and significant motor challenges. Here, we studied the developmental dynamics of a subpopulation of cortical neurons co-expressing CTIP2 and BRN1. We found that CTIP2+BRN1+ neurons are born during early phases of neurogenesis like other CTIP2+ neurons, peak in expression during perinatal life, and persist in adult brains. We also found that CTIP2+BRN1+ neurons are excessive in number in prenatal and mature cortical motor areas of *Ddx3x* mutant mice, translating into altered laminar distribution of subcerebral projection neurons extending axons to the brainstem. These findings underscore the critical role of molecular specification during cortical development in health and disease.

## Teaser

**Summary:** A subpopulation of cortical neurons is altered in a mouse model of DDX3X syndrome.

## INTRODUCTION

The mammalian cerebral cortex is organized in a six-layered cytoarchitecture that hosts glutamatergic projection neurons with distinct long-range connectivity patterns (Greig et al., 2013; Lodato and Arlotta, 2015). Neurons residing in deep layers (DL) form mostly corticofugal projections extending to the thalamus (layer VI corticothalamic projection neurons, CthPNs) or to the striatum, brainstem, and spinal cord (layer V subcerebral projection neurons, ScPNs). On the contrary, intra-telencephalic projection neurons (ITNs) in either deep or upper layers (UL) extend their axons to the ipsilateral or contralateral telencephalic regions, the latter mostly crossing the midline through the corpus callosum (callosal projection neurons, CPNs). The genesis of these neuronal subtypes unfolds in an inside-out manner: DL neurons are predominantly born during early phases of cortical neurogenesis (between embryonic day, E, 11.5 and 13.5 in mouse), while UL neurons are born at a later stage (E14.5-16.5) and migrate past the DL neurons to occupy the more superficial layers (Hevner et al., 2001; Lai et al., 2008; Magrinelli et al., 2022).

The molecular identity of these neuronal subtypes is largely dictated by complex regulatory networks defined by transcription factors (TFs) and chromatin remodelers, for example BRN1/*Pou3f3* for UL ITNs, SATB2/*Satb2* for UL or DL CPNs, CTIP2/*Bcl11b* or FEZL/*Fezf2* for DL ScPNs, and TBR1/*Tbr1*, TLE4/*Tle4*, or FOXP2/*Foxp2* for DL CthPNs (Alcamo et al., 2008; Arlotta et al., 2005; Baranek et al., 2012; Barão et al., 2024; Bedogni et al., 2010; Britanova et al., 2005; Cánovas et al., 2015; Chen et al., 2008; Dominguez et al., 2013; Fazel Darbandi et al., 2018; Galazo et al., 2016, 2023; Leone et al., 2015; McKenna et al., 2011; Molyneaux et al., 2005; Molyneaux et al., 2015; Oishi et al., 2016; Srivatsa et al., 2014; Sugitani et al., 2002). Co-expression of TFs driving distinct identities has been documented as a transient developmental event that fades because of progressive TFs cross-regulation (McKenna et al., 2011) or as the result of post-mitotic resolution during subtype differentiation (Azim et al., 2009; Kwan et al., 2008). Further, subpopulations co-expressing antithetic TFs and persisting postnatally [e.g., CTIP2+SATB2+ neurons (Harb et al., 2016)] or with dual projection targets (Cederquist et al., 2013; Galazo et al., 2016, 2023; Mitchell and Macklis, 2005; Veinante and Deschênes, 2003) have been reported. Additional complexity arises from refinement of neuron subtype identity with the acquisition of area-specific properties (e.g. motor versus sensory) during cortical parcellation (Abe et al., 2024; Bhaduri et al., 2021; Galazo et al., 2016; Harb et al., 2016; Nowakowski et al., 2017; Tomassy et al., 2010).

The orchestration of cell fate decisions during cortical development is critical for the formation of circuits subserving complex behaviors (Boitnott et al., 2021; Fang et al., 2014; Fazel Darbandi et al., 2018) and alterations in the balance of neuronal populations have been reported in cellular or animal models of neurodevelopmental disorders (NDDs) (Bedogni et al., 2010; Cánovas et al., 2015; Jin et al., 2020; Li et al., 2023; Panagiotakos et al., 2019; Paulsen et al., 2022). Coherently, mutations in *POU3F3* (Snijders Blok et al., 2019), *SATB2* (Bengani et al., 2017; Fu et al., 2022; Leoyklang et al., 2007), *BCL11B* (Lessel et al., 2018), or *TBR1* (De Rubeis et al., 2014; Deriziotis et al., 2014) are associated with NDDs, including intellectual disability (ID) and autism spectrum disorder (ASD).

We have recently discovered alterations in the molecular specification of cortical glutamatergic neurons in a mouse model of DDX3X syndrome (Boitnott et al., 2021), an X-linked neurodevelopmental disorder that primarily affects females and is caused by mutations in the DEAD box helicase 3 x-linked gene (*DDX3X*) (Lennox et al., 2020; Snijders Blok et al., 2015; von Mueffling et al., 2024). Mutations in *DDX3X* are a high-risk factor for ASD co-morbid with ID in females (Ruzzo et al., 2019; Turner et al., 2019). Affected individuals can also be diagnosed with congenital brain malformations suggestive of defective corticogenesis (Lennox et al., 2020; Snijders Blok et al., 2015; Tang et al., 2021). Consistent with these clinical observations, studies in mouse models have shown that *Ddx3x* orchestrates cortical neurogenesis and lamination (Boitnott et al., 2021; Hoye et al., 2022; Lennox et al., 2020). Specifically, we have previously found that *Ddx3x* haploinsufficient (*Ddx3x*^+/−^) female mice at postnatal (P) day 3 display alterations of CTIP2-expressing neurons in layer V. These alterations include a surplus of neurons co-expressing the antithetic TFs CTIP2 and BRN1 (CTIP2+BRN1+ neurons) and are specific to motor areas (Boitnott et al., 2021).

Virtually nothing is known about this minority of layer V CTIP2+BRN1+ neurons even in physiological conditions, including their time of birth and whether they represent a transient population. Seeking to elucidate these aspects, we first followed their temporal dynamics at prenatal, postnatal, juvenile, and adult time points in control mice, and their genesis during embryonic development. We then sought to assess whether the effect of *Ddx3x* mutations on this subpopulation precedes birth and perdures in adulthood to affect circuits established by ScPNs.

We found that CTIP2+BRN1+ neurons are born during deep-layer neurogenesis like other CTIP2+ neurons, persist in the adult cortex, and their temporal dynamics differ by cortical area. We also found that *Ddx3x* haploinsufficiency results in increased co-expression of CTIP2 and BRN1 in embryonic post-migratory neurons and layer V adult neurons in motor areas, which translates into altered ScPNs connecting the cortex with the brainstem.

## RESULTS

### Cortical subpopulations co-expressing TFs CTIP2 and BRN1 change over the course of development

During cortical development, the TF CTIP2 is expressed in post-mitotic neurons in the cortical plate (CP), as opposed to progenitors in the ventricular zone (VZ) and subventricular zone (SVZ), and then progressively drives the fate of ScPNs within layer V (Arlotta et al., 2005; Chen et al., 2008; McKenna et al., 2011). A subgroup of CTIP2+ neurons co-expressing BRN1/2 have been documented in the CP as early as E15.5 (Dominguez et al., 2013). Further, a subpopulation of CTIP2+ co-expressing SATB2 emerges at birth (Harb et al., 2016).

**Fig. 1.**
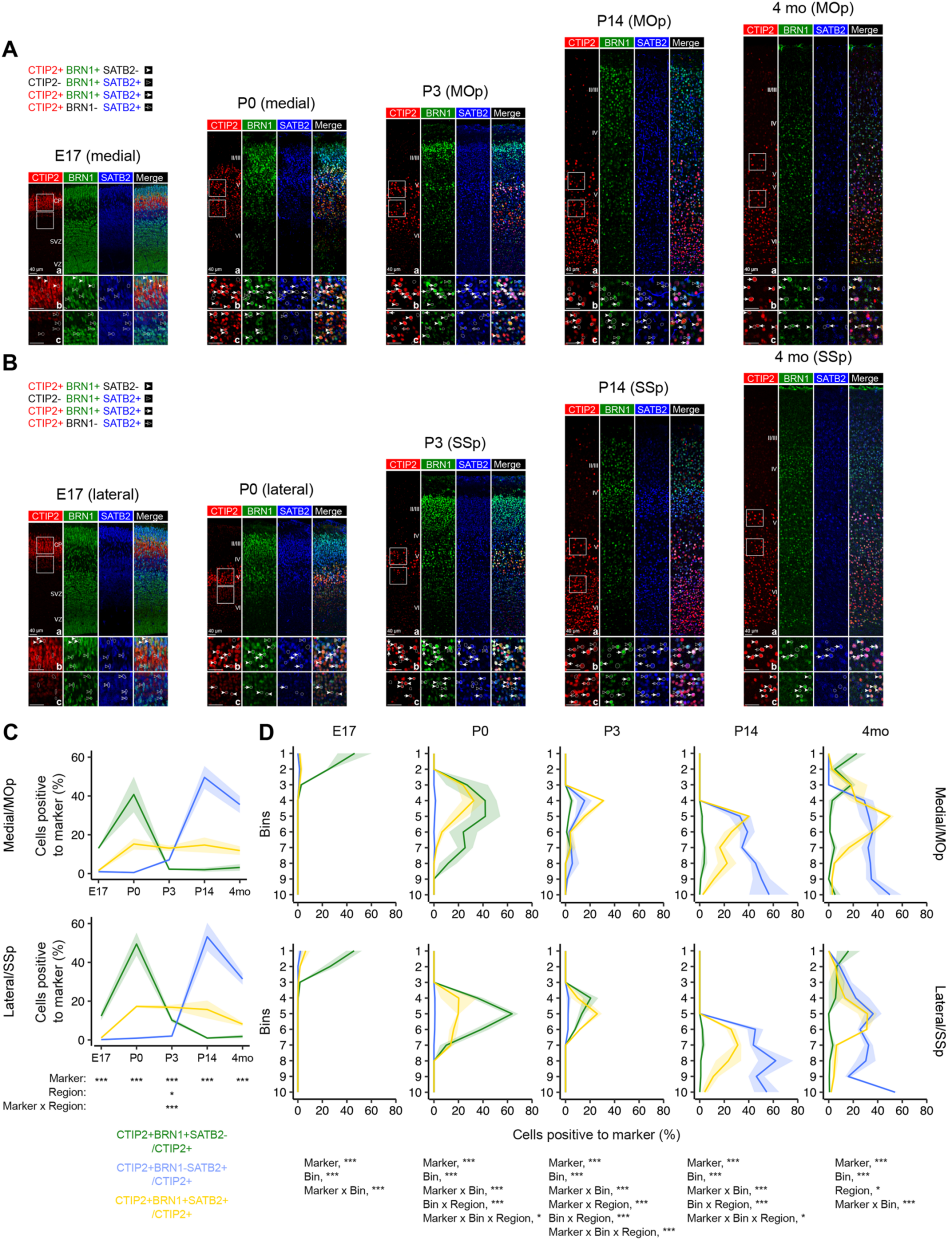
**Subpopulations of layer V neurons change dynamically during cortical development.** (A) Confocal images of medial cortex and mature primary motor cortex (MOp) immunostained for CTIP2 (red), BRN1 (green), and SATB2 (blue) at embryonic day (E) 17, postnatal (P) day 0, P3, P14 or 4 months. (B) Confocal images of lateral cortex and mature primary somatosensory cortex (SSp) immunostained for CTIP2 (red), BRN1 (green), and SATB2 (blue) at E17, P0, P3, P14 or 4 months. Panels in rows b and c show magnifications of the insets in panels in row a. Images are representative of at least *n*=3 mice/experiment. CP, cortical plate; E, embryonic day; MOp, primary motor cortex; P, postnatal day; SSp, primary somatosensory cortex; SVZ, subventricular zone; VZ, ventricular zone. Empty arrows: CTIP2+BRN1−SATB2+; full arrows: CTIP2+BRN1+SATB2+; empty arrowheads: CTIP2−BRN1+SATB2+; full arrowheads: CTIP2+BRN1+SATB2**-**. (C) Percentage of CTIP2+BRN1+SATB2− (green), CTIP2+BRN1−SATB2+ (blue), and CTIP2+BRN1+SATB2+ (yellow) over all CTIP2+ cells across the developmental stages indicated in A and B and in both medial/MOp (upper panel) and lateral/SSp (lower panel) regions of the cortex. *n*=3 mice/experiment; mean±s.e.m.; ANOVA for marker (*P*-value<0.001), region (not significant), developmental stage (*P*-value<0.001), and their interactions (only marker x stage significant, *P*-value<0.001), followed by two-way ANOVA comparing marker and region within each stage, as indicated below the plots; *P-value<0.05, ***P-value<0.001. (D) Laminar distribution of the percentage of CTIP2+BRN1+SATB2− (green), CTIP2+BRN1−SATB2+ (blue), and CTIP2+BRN1+SATB2+ (yellow) cells over all CTIP2+ cells across the developmental stages indicated in A and B and in both medial/MOp (upper panel) and lateral/SSp (lower panel) regions of the cortex, from the pia (Bin 1) to the ventricle (Bin 10). *n*=3 mice/experiment; mean±s.e.m.; multi-way ANOVA for marker, region, bins, developmental stage, and their interactions (only significant variables shown below the plots); *P-value<0.05, **P-value<0.001, ***P-value<0.001.

We sought to delineate the spatial and temporal dynamics of CTIP2, BRN1, and SATB2 co-expression to map the CTIP2+BRN1+ subpopulation and assess its specificity compared to the pool of CTIP2 neurons expressing SATB2. To this end, we performed immunostainings for CTIP2, BRN1, and SATB2 in the medial/primary motor (MOp) and lateral/primary somatosensory (SSp) areas of the cortex at embryonic day (E) 17 (after the peak of UL neurogenesis), birth (P0), postnatal day 3 (P3), two weeks (P14), and 4 months (4 mo) (Fig. 1A-B). We then quantified the abundance and laminar distribution of CTIP2+BRN1+ and CTIP2+SATB2+ neurons (Fig. S1A-B). To separate each subpopulation, we further quantified CTIP2+BRN1+ neurons that did not express SATB2 (CTIP2+BRN1+SATB2−) and CTIP2+SATB2+ neurons that did not express BRN1 (CTIP2+BRN1-SATB2+). The overlap between subpopulations was also quantified (CTIP2+BRN1+SATB2+) (Fig. 1C-D). We found that the abundance of these populations dramatically changed as development progressed, with CTIP2+BRN1+SATB2− neurons peaking at birth, CTIP2+BRN1−SATB2+ reaching highest abundance at P14, and CTIP2+BRN1+SATB2+ neurons maintaining stable numbers over time (Fig. 1C, Fig. S1A, see also legend).

At E17, CTIP2 marks post-mitotic neurons in the CP, SATB2 is expressed in newborn UL neurons migrating past CTIP2+ neurons or already in the CP, and BRN1 labels both neurons in the CP and progenitors in the VZ and SVZ (Fig. 1A-B, panels a), in line with previous observations (Arlotta et al., 2005; Britanova et al., 2005; Dominguez et al., 2013; Sugitani et al., 2002). In the prospective DL of the CP (Fig. 1A-B, panels b), we detected CTIP2+BRN1+ or BRN1+SATB2+ neurons, but no CTIP2+SATB2+ neurons (Fig. 1C-D), consistent with prior reports (Dominguez et al., 2013; Harb et al., 2016). BRN1+SATB2+ neurons were also observed in the subplate and intermediate zone (Fig. 1A-B, panels c).

The dynamics of these subpopulations drastically changed perinatally, when region-specific patterns also begun to emerge (Fig. 1C). At P0, CTIP2+ neurons delineated a broader and denser layer V in the medial (prospective motor) compared to the lateral (prospective sensory) cortex (Fig. 1A-B,D), as expected (Harb et al., 2016; Tomassy et al., 2010). In addition to CTIP2+BRN1+ and BRN1+SATB2+ neurons, CTIP2+BRN1+SATB2+ neurons also appeared (Fig. 1A-B,D). Importantly, almost all the CTIP2+SATB2+ neurons at this stage were also expressing BRN1 (Fig. 1C-D). Further, CTIP2+ subpopulations varied in laminar distribution based on the cortical area, with CTIP2+BRN1+SATB2− neurons showing a more compact laminar position in the lateral cortex compared to the medial cortex, and in the medial cortex CTIP2+BRN1+SATB2+ neurons being more abundant in superficial layer V compared to CTIP2+BRN1+SATB2− neurons (Fig. 1A-B,D).

Between P0 and P3, the percentage of CTIP2+ neurons expressing both SATB2 and BRN1 remained comparable, but the abundance of those co-expressing only BRN1 dropped as the number of those co-expressing only SATB2 begun to increase (Fig. 1A-C). The relative proportions differed by cortical area, as there were more CTIP2+ neurons co-expressing only BRN1 in the developing somatosensory cortex than the motor cortex (Fig. 1A-D). At P14, the percentage of CTIP2+ neurons expressing only SATB2 peaked, as the CTIP2+SATB2+BRN1+ pool remained stable (Fig. 1A-D). These two CTIP2 subpopulations (CTIP2+SATB2+BRN1+ and CTIP2+BRN1−SATB2+) persisted in adulthood (Fig. 1; Fig. S1).

These data indicate that the subpopulation of CTIP2+ neurons co-expressing BRN1 first appears during embryonic development in post-migratory neurons, changes early postnatally, and persists through adulthood. The CTIP2+BRN1+ neurons are, to some extent, distinct from the CTIP2+SATB2+ pool.

### CTIP2+BRN1+ neurons are born during early neurogenesis

To better define the nature of the CTIP2+BRN1+ neurons, we sought to track their genesis. To this end, we performed cell ‘birth-dating’ experiments with a single prenatal pulse of the synthetic thymidine analogue 5-bromo-2′-deoxyuridine (BrdU), which intercalates into the DNA during replication. We intraperitoneally administered 50 mg/kg BrdU to E13.5 or E16 pregnant females to capture DL and UL neurogenesis, respectively. We then examined the proportion of CTIP2+, BRN1+, or CTIP2+BRN1+ cells positive to BrdU in the medial and lateral cortices of their P3 offspring.

**Fig. 2.**
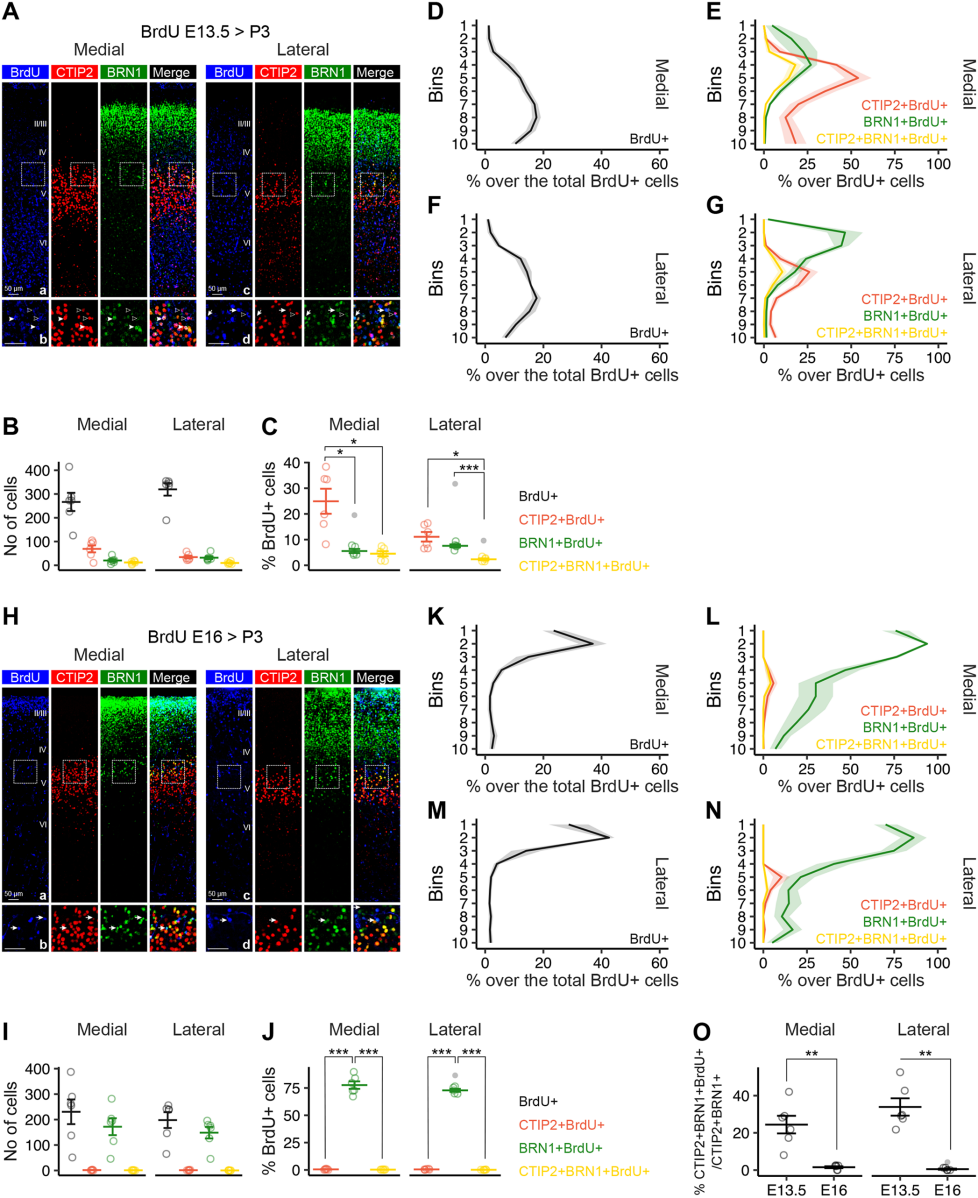
**Cortical CTIP2+BRN1+ neurons are born during the early wave of neurogenesis.** (A-G) Molecular identity and laminar distribution of E13.5-born neurons in P3 cortices via *in utero* BrdU labeling. (A) Confocal images of medial (a-b) and lateral (c-d) cortices immunostained for BrdU (blue), CTIP2 (red), BRN1 (green) isolated from the P3 offspring of pregnant dams injected with BrdU at E13.5. Panels in rows b and d show magnifications of the insets in panels in rows a and c, respectively. Empty arrowheads, CTIP2+BrdU+; full arrowheads, CTIP2+BRN1+ BrdU+; full arrows, BRN1+BrdU+. (B) Number of all BrdU+ (black), BrdU+ CTIP2+ (red), BRN1+BrdU+ (green), or CTIP2+BRN1+BrdU+ (yellow) cells in medial and lateral cortices from the experiment in A; mean±s.e.m. (C) Percentage of BrdU+CTIP2+ (red), BrdU+BRN1+ (green), or BrdU+CTIP2+BRN1+ (yellow) over the total of BrdU+ cells in medial and lateral cortices from the experiment in A. *n*=6 mice/experiment; mean±s.e.m.; statistical testing was conducted after removing outliers (indicated as grey data points) and assessing normality with the Shapiro–Wilk test; two-way ANOVA for region (*P*-value<0.05), marker (*P*-value<0.001), and their interaction (*P*-value<0.01), followed by Student's *t*-test with Benjamini–Hochberg *post hoc* correction, as shown in the plot; *P-value<0.05, ***P-value<0.001. (D) Laminar distribution of E13.5-born cells in the medial cortex, as shown by the percentage of BrdU+ cells in each of 10 bins from the pia (Bin 1) to the ventricle (Bin 10). (E) Laminar distribution of E13.5-born cells positive to CTIP2 (red), BRN1 (green), or both (yellow) in the medial cortex, as shown by the percentage of CTIP2+BrdU+ (red), BRN1+BrdU+ (green), or CTIP2+BRN1+BrdU+ (yellow) cells in each of 10 bins from the pia (Bin 1) to the ventricle (Bin 10). (F) As in D, but in the lateral cortex. Comparing with panel D, two-way ANOVA for bins (*P*-value<0.001), region (not significant), and their interaction (*P*-value<0.05). (G) As in E, but in the lateral cortex. Comparing with panel E, multiple-way ANOVA for bins (*P*-value<0.001), region (*P*-value<0.001), marker (*P*-value<0.001), and their interaction (*P*-value<0.001). (H-N) Molecular identity and laminar distribution of E16-born neurons in P3 cortices via *in utero* BrdU labeling. (H) Confocal images of medial (a-b) and lateral (c-d) cortices immunostained for BrdU (blue), CTIP2 (red), BRN1 (green) isolated from the P3 offspring of pregnant dams injected with BrdU at E16. Panels in rows b and d show magnifications of the insets in panels in rows a and c, respectively. Empty arrowheads, CTIP2+BrdU+; full arrowheads, CTIP2+BRN1+BrdU+; full arrows, BRN1+BrdU+. (I) Number of all BrdU+ (black), CTIP2+BrdU+ (red), BRN1+BrdU+ (green), or CTIP2+BRN1+BrdU+ (yellow) cells in medial and lateral cortices from the experiment in H. mean±s.e.m. (J) Percentage of CTIP2+BrdU+ (red), BRN1+BrdU+ (green), or CTIP2+BRN1+BrdU+ (yellow) over the total of BrdU+ cells in medial and lateral cortices from the experiment in H. *n*=6 mice/experiment; mean±s.e.m.; statistical testing was conducted after removing outliers (indicated as grey data points) and assessing normality with the Shapiro–Wilk test; two-way ANOVA for region (*P*-value<0.001), marker (*P*-value<0.001), and their interaction (not significant), followed by Student's *t*-test with Benjamini–Hochberg *post hoc* correction, as shown in the plot; ***P-value<0.001. (K) Laminar distribution of E16-born cells in the medial cortex, as shown by the percentage of BrdU+ cells in each of 10 bins from the pia (Bin 1) to the ventricle (Bin 10). (L) Laminar distribution of E16-born cells positive to CTIP2, BRN1, or both in the medial cortex, as shown by the percentage of CTIP2+BrdU+ (red), BRN1+BrdU+ (green), or CTIP2+BRN1+BrdU+ (yellow) cells in each of 10 bins from the pia (Bin 1) to the ventricle (Bin 10). (M) As in K, but in the lateral cortex. Comparing with panel K, two-way ANOVA for bins (*P*-value<0.001), region (not significant), and their interaction (not significant). (N) As in L, but in the lateral cortex. Comparing with panel L, multiple-way ANOVA for bins (*P*-value<0.001), region (*P*-value<0.05), marker (*P*-value<0.001), and all their interactions (marker×bins, *P*-value<0.001; marker×region, *P*-value<0.05; all other interactions, not significant). (O) Percentage of CTIP2+BRN1+ cells born at E13.5 or E16 in medial or lateral cortices as in the experiments in panels A-G and H-N respectively, estimated as ratio of CTIP2+BRN1+BrdU+ cells over all CTIP2+BRN1+ cells. *n*=6 mice/experiment/time point; mean±s.e.m.; statistical testing was conducted after removing outliers (indicated as grey data points) and assessing normality with the Shapiro–Wilk test; Wilcoxon signed-rank test, **P-value<0.01.

In line with previous evidence (Hoerder-Suabedissen and Molnár, 2013; Magrinelli et al., 2022), date of birth determines radial position more sharply as corticogenesis advances. In fact, E13.5-born neurons display great radial dispersion (Fig. 2A,D,F), while E16-born neurons are laminarly compact (Fig. 2H,K,M) in P3 cortices. As expected, genesis of layer V CTIP2+ neurons is abundant during early phases of neurogenesis (Fig. 2A-G) and completed by E16 (Fig. 2H-J), while the birth of UL BRN1+ neurons is robust at E16 (Fig. 2H-N).

Further, in line with the reported asynchronicity of maturation of the medial and lateral cortices (Hoerder-Suabedissen and Molnár, 2013; Keil et al., 2020), we found area-specific differences in the timing of birth of these neuronal populations during early phases of neurogenesis (Fig. 2A-C). In fact, over 20% of neurons born at E13.5 and surviving until P3 are CTIP2+ in the medial cortex (Fig. 2A-C) and E13.5-born neurons constitute ∼50% of those that will populate layer V (Fig. 2A,D-E). In the lateral cortex instead, the birth of CTIP2+ neurons has largely progressed by E13.5 (Fig. 2A-C), and the birth of UL neurons has picked up, as shown by the distribution of E13.5-born BRN1+ neurons at P3 (Fig. 2A,F-G).

As for the CTIP2+BRN1+ subpopulation, 20-30% of those existing at P3 were born at E13.5 and virtually none were born at E16 (Fig. 2O,J). In keeping with the general underrepresentation of these neurons at P3 (Fig. 1), the overall number of CTIP2+BRN1+ cells over all those born at E13.5 is low (Fig. 2B-C).

Overall, these data indicate that CTIP2+BRN1+ neurons originate during DL neurogenesis comparable with other CTIP2+ neurons.

### *Ddx3x* haploinsufficiency alters the balance of CTIP2+BRN1+ neurons during development

Our previous work has shown an alteration of DL and UL neurons in *Ddx3x*^+/−^ P3 pups, with area-specific differences (Boitnott et al., 2021). Compared with their *Ddx3x*^+/+^ littermate controls, P3 *Ddx3x*^+/−^ pups displayed more CTIP2+ neurons but comparable number of BRN1+ neurons in the developing MOp, with no changes in CTIP2+ neurons and more BRN1+ neurons in the SSp (Boitnott et al., 2021). The CTIP2+ neurons of *Ddx3x*^+/−^ MOp also displayed disproportional distribution in deeper strata of layer V, and higher mixed BRN1+ identity (Boitnott et al., 2021). No changes in CTIP2+ laminar distribution or CTIP2+BRN1+ population was observed in the SSp (Boitnott et al., 2021).

**Fig. 3.**
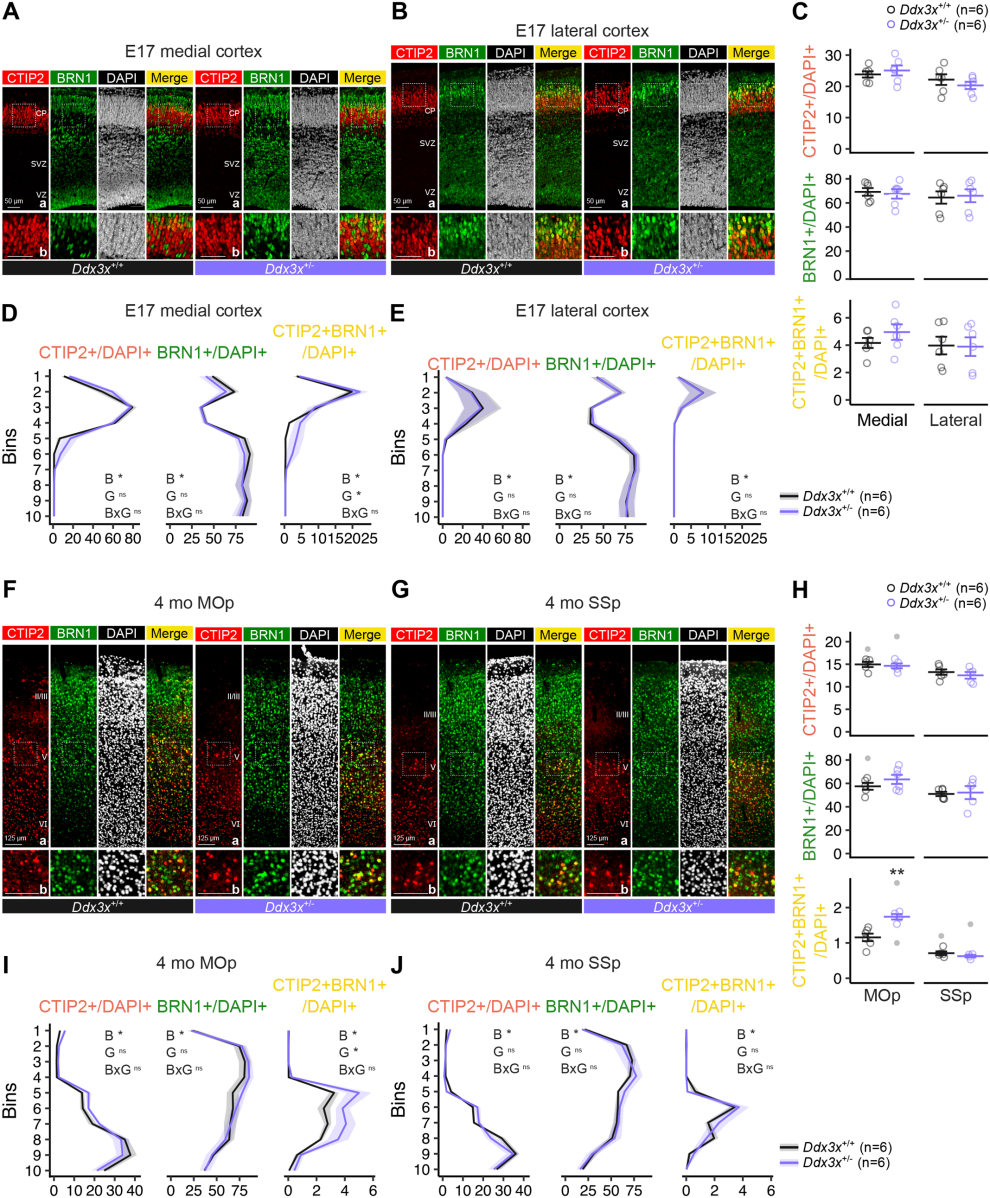
***Ddx3x* mutant female mice have an excess of the CTIP2+BRN1+ subpopulation in the motor cortex starting prenatally and persisting in adulthood.** (A) Confocal images of immunostaining for CTIP2 (red), BRN1 (green), and DAPI (white) on the medial cortex from E17 *Ddx3x*^+/−^ female embryos (lavender) or control *Ddx3x*^+/+^ control female embryos (black). CP, cortical plate; VZ, ventricular zone; SVZ, subventricular zone. Panels in row b show magnifications of the insets in row a. (B) As in A, but for the lateral cortex. (C) Quantifications of the percentage of CTIP2+ (red), BRN1+ (green) or CTIP2+BRN1+ (yellow) over the total cells (DAPI+) on the medial and lateral cortices from E17 *Ddx3x*^+/−^ female embryos (lavender) or control *Ddx3x*^+/+^ control female embryos (black) as in A and B. *n*=6 mice/genotype/region; mean±s.e.m. (D) Laminar distribution of the CTIP2+ (red), BRN1+ (green) or CTIP2+BRN1+ (yellow) over the total cells (DAPI+) in medial cortices from E17 *Ddx3x*^+/−^ female embryos (lavender) or control *Ddx3x*^+/+^ control female embryos (black) as in A. *n*=6 mice/genotype; mean±s.e.m.; two-way ANOVA for bins (B), genotype (G), and their interaction (B×G); *P-value<0.05. (E) As in D but for the lateral cortex. (F) Confocal images of immunostaining for CTIP2 (red), BRN1 (green), and DAPI (white) on the MOp from 4-month-old *Ddx3x*^+/−^ female mice (lavender) or control *Ddx3x*^+/+^ control female mice (black). Panels in row b show magnifications of the insets in a. (G) As in F, but for the SSp. (H) Quantifications of the percentage of CTIP2+ (red), BRN1+ (green) or CTIP2+BRN1+ (yellow) over the total cells (DAPI+) on the MOp and SSp from from 4-month-old *Ddx3x*^+/−^ female mice (lavender) or control *Ddx3x*^+/+^ control female mice (black), as in F and G. *n*=6 mice/genotype/region; mean±s.e.m.; statistical testing was conducted after removing outliers (indicated as grey data points) and assessing normality with the Shapiro–Wilk test; Student's *t*-test, as shown in the plot; *P-value<0.05. (I) Laminar distribution of the CTIP2+ (red), BRN1+ (green) or CTIP2+BRN1+ (yellow) over the total cells (DAPI+) in the MOp of 4-month-old *Ddx3x*^+/−^ female mice (lavender) or control *Ddx3x*^+/+^ control female mice (black) as in F. *n*=6 mice/genotype; mean±s.e.m.; two-way ANOVA for bins (B), genotype (G), and their interaction (B×G); *P-value<0.05. (J) As in I, but for the SSp. MOp, primary motor cortex; P, postnatal day; SSp, primary somatosensory cortex.

Based on these observations, we sought to characterize the developmental impact of *Ddx3x* mutations on the CTIP2+BRN1+ population. To this end, we performed immunostainings for CTIP2 and BRN1 at E17, a prenatal time point after the birth of CTIP2+BRN1+ neurons (Fig. 2), and in adult cortices (4-months-old) from *Ddx3x*^+/−^ and control *Ddx3x*^+/+^ mice. We found that the excess of CTIP2+BRN1+ neurons first appears beneath the CP of the medial (but not lateral) cortex at E17 (Fig. 3A-E). This indicates that the increased co-expression of CTIP2 and BRN1 in *Ddx3x*^+/−^ mutant brains is already present in post-migratory neurons. The regional specificity of this effect might depend upon earlier latero-medial graded neurogenesis and/or the reported latero-medial gradient in BRN1 expression (Dominguez et al., 2013).

We also found that this regional specificity persists in adulthood, as the MOp (but not the SSp) of 4-month-old *Ddx3x*^+/−^ females shows a surplus of CTIP2+BRN1+ neurons (Fig. 3F-J). The vulnerability of the motor areas is consistent with the motor delays and adult motor deficits displayed by *Ddx3x*^+/−^ mice (Boitnott et al., 2021).

These data corroborate that *Ddx3x* mutations affect the density of the neurons co-expressing TFs CTIP2 and BRN1 starting prenatally and continuing in adulthood, with specificity for the developing and adult motor areas of the cortex.

### Innervation targets of CTIP2+BRN1+ neurons

CTIP2 instructs neurons to extend subcerebral projections (Arlotta et al., 2005; Chen et al., 2008; Greig et al., 2013; Harb et al., 2016). Given that the surplus of CTIP2+ neurons co-expressing BRN1 in *Ddx3x*^+/−^ brains is restricted to motor areas (Fig. 3) and the evidence of motor delays and deficits in *Ddx3x*^+/−^ mice (Boitnott et al., 2021), we asked whether ScPNs engaged in motor circuits might be impacted by *Ddx3x* mutations.

Among the diverse subtypes of ScPNs (Lodato and Arlotta, 2015), we prioritized cortico-brainstem projections reaching the pons, which form a major relay of corticocerebellar communication critical for motor control (e.g. Gao et al., 2018). This choice was guided by evidence that individuals with *DDX3X* mutations can have pontine hypoplasia (Lennox et al., 2020; Scala et al., 2019) and/or cerebellar malformations (Aldinger et al., 2019; Lennox et al., 2020) and that motor delays and dysfunctions are pervasive in patients with DDX3X syndrome (Lennox et al., 2020; Levy et al., 2023; Snijders Blok et al., 2015).

**Fig. 4.**
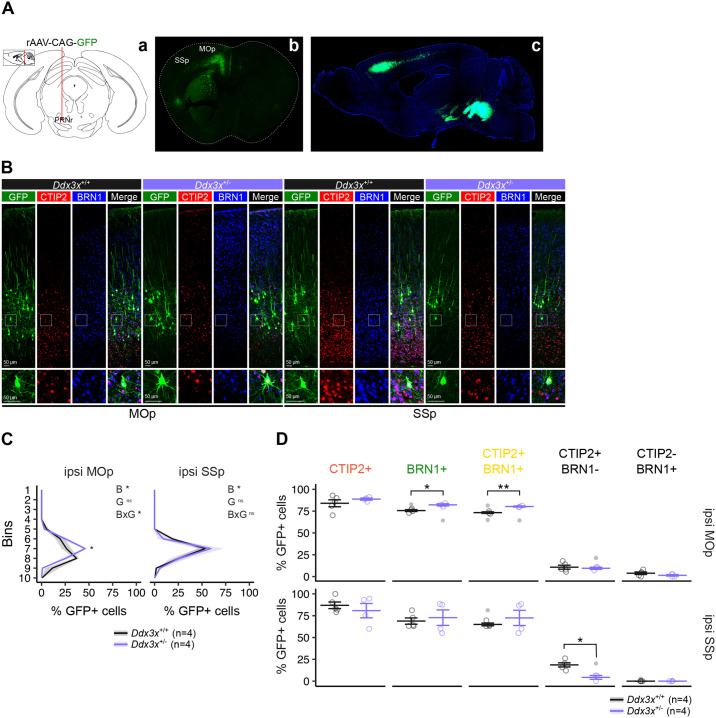
**Cortico-brainstem neurons of *Ddx3x* mutant female mice have altered laminar position and expression of CTIP2 and BRN1.** (A) Retrograde tracing of cortico-brainstem neurons reaching the pons. Subpanel a, schematics of the coronal plane corresponding to the site of injection of rAVV-CAG-GFP (pontine reticular nucleus, PNRr), including a sagittal view. Subpanel b, coronal section of a brain 3 weeks after the stereotaxic surgery, showing the ipsilateral labeling of neurons extending axons from the MOp and SSp to the brainstem. Subpanel c, sagittal section of a brain 3 weeks after the stereotaxic surgery, as in subpanels a and b. (B) Representative confocal images of immunostaining for GFP (green), CTIP2 (red), BRN1 (blue) on MOp and SSp isolated from 8-week-old *Ddx3x*^+/−^ female mice (lavender) or control *Ddx3x*^+/+^ female mice (black) injected 3 weeks prior with rAVV-CAG-GFP in the PNRr, as in A. (C) Laminar distribution of cortico-brainstem neurons in the ipsilateral MOp and SSp of *Ddx3x*^+/−^ female mice (lavender) or control *Ddx3x*^+/+^ female mice (black), as shown by the percentage of GFP+ somata in each of 10 bins from the pia (Bin 1) to the ventricle (Bin 10) over the absolute number of GFP+ somata. *n*=4 mice/genotype; mean±s.e.m.; multiple-way ANOVA comparing data for MOp and SSp for bins (*P*-value<0.05), genotype (not significant), region (not significant), and all their interactions (all not significant, with the exception of bins×region, *P*-value<0.05); within each region, two-way ANOVA for bins (B), genotype (G), and their interaction (B×G), followed by Student's *t*-test with Benjamini–Hochberg *post-hoc* correction for the ipsilateral MOp, as shown in the plot; *P-value<0.05. (D) Expression of CTIP2 and BRN1 in cortico-brainstem neurons in the ipsilateral MOp and SSp of *Ddx3x*^+/−^ female mice (lavender) or control *Ddx3x*^+/+^ female mice (black), as quantified by the percentage of GFP+ somata also expressing CTIP2, BRN1, CTIP2 and BRN1, CTIP2 but not BRN1, or BRN1 but not CTIP2, over the absolute number of GFP+ somata. *n*=4 mice/genotype; mean±s.e.m.; statistical testing was conducted after removing outliers (shown as grey data points) and assessing normality with the Shapiro–Wilk test; Student's *t*-test, as shown in the plot; *P-value<0.05, **P-value<0.01. MOp, primary motor cortex; SSp, primary somatosensory cortex.

To label cortico-brainstem neurons, we performed retrograde tracing experiments by injecting rAVV-CAG-GFP in the pons of 5-week-old *Ddx3x*^+/−^ and control *Ddx3x*^+/+^ female mice, resulting in the robust labeling of the ipsilateral axonal tracts and somata 3 weeks post-surgery (Fig. 4A). These neurons form mostly ipsilateral projections (Henschke and Pakan, 2020) that can terminate in the pons or extend to the spinal cord via the pyramidal tract (Muñoz-Castañeda et al., 2021; Nelson et al., 2021). First, we examined the radial distribution of GFP+ somata in the ipsilateral MOp and SSp, which reside in layer Vb, as previously reported (Kim et al., 2015; Moya et al., 2022; Muñoz-Castañeda et al., 2021). In line with previous observations (Moya et al., 2022), the somata of the GFP+ neurons are distributed in a broader band in the MOp compared to the SSp, as indicated by a statistically significant interaction between region and bin (*P*-value<0.05) (Fig. 4B-C, see also legend). While the laminar distribution of GFP+ somata in SSp appears comparable across genotypes, the broader band of GFP+ somata in the MOp is no longer seen in *Ddx3x*^+/−^ female mice, as shown by the statistically significant increase in GFP+ neurons in bin 7 only in the MOp (Fig. 4B-C). This evidence is consistent with our other data pointing at a greater vulnerability of motor areas to *Ddx3x* mutations (Fig. 3).

Second, we calculated the percentage of GFP+ neurons expressing CTIP2, BRN1, CTIP2 and BRN1, CTIP2 but not BRN1, or BRN1 but not CTIP2 (Fig. 4D). We found that the portion of cortico-brainstem neurons residing in the MOp and expressing BRN1 or both CTIP2 and BRN1 is excessive in *Ddx3x*^+/−^ female mice compared to controls (Fig. 4D), in keeping with the surplus of CTIP2+BRN1+ cells in the MOp of adult *Ddx3x*^+/−^ female mice (Fig. 3). We also found that SSp cortico-brainstem neurons expressing solely CTIP2 (and not BRN1) are largely lost in *Ddx3x*^+/−^ female mice (Fig. 4D).

All together, these results indicate that *Ddx3x* deletion alters cortico-brainstem neurons in the MOp in their laminar distribution and expression of the identity-driving TFs CTIP2 and BRN1. These cellular alterations might contribute to the delays in meeting motor milestones and adult motor deficits seen in *Ddx3x*^+/−^ mutant mice (Boitnott et al., 2021).

## DISCUSSION

Cortical neuronal diversity is largely shaped by mutually repressive TFs driving opposite molecular identities and guiding intra- and extra-telencephalic projection patterns (Greig et al., 2013; Lodato and Arlotta, 2015). However, subpopulations of neurons co-expressing antithetic TFs that persist in adulthood have been reported, for example layer V neurons co-expressing CTIP2 and SATB2 emerging postnatally and progressively enriching in somatosensory areas (Harb et al., 2016). Here, we provide evidence for a subpopulation of layer V neurons co-expressing TFs CTIP2 and BRN1. These neurons are born during early waves of neurogenesis along with the rest of the layer V CTIP2+ pool (Fig. 2). CTIP2+BRN1+ peak in number around birth and persist in adulthood (Fig. 1; Fig. S1). These CTIP2+BRN1+ neurons are partially distinct from the previously reported CTIP2+SATB2+ neurons, as the abundant CTIP2+BRN1+ neurons detected perinatally are mostly negative to SATB2 (Fig. 1). These developmental dynamics follow area-specific patterns (Fig. 1), which is not surprising considering the molecular identity of motor (e.g., Joshi et al., 2008; Sun et al., 2005) and sensory (e.g., Greig et al., 2016) areas.

We also extend on our earlier findings of altered balance of these CTIP2+BRN1+ neurons in brains with *Ddx3x* haploinsufficiency (Fig. 3), which in humans is associated with a complex NDD manifesting with ID and ASD (Lennox et al., 2020; Levy et al., 2023; Snijders Blok et al., 2015). We show that the excess of layer V neurons co-expressing CTIP2 and BRN1 in *Ddx3x* haploinsufficient cortices appears already prenatally in post-migratory neurons and perdures into adulthood (Fig. 3). Notably, this excess is restricted to the motor area of the cortex (Fig. 3) and reflects on corticofugal projections from the motor areas to the brainstem (Fig. 4). These cellular phenotypes go hand in hand with our earlier findings that *Ddx3x* mutant mice have postnatal hypotonia and motor delays that translate into adult motor deficits (Boitnott et al., 2021), reminiscent of the motor delays and deficits observed in the patient population (Lennox et al., 2020; Snijders Blok et al., 2015; Tang et al., 2021). Framed in the larger context, these findings add on the evidence of altered cortical populations in mouse models with mutations in orthologues of NDD risk genes (Bedogni et al., 2010; Cánovas et al., 2015; Usui et al., 2017; Woodworth et al., 2016) and orthogonal genomic data corroborating that the prenatal development of the cortex is a critical window of vulnerability to ASD (Fu et al., 2022; Polioudakis et al., 2019; Willsey et al., 2013).

Previous studies have shown that *Ddx3x* is required for progenitor proliferation and neuronal differentiation in the developing cortex (Hoye et al., 2022; Lennox et al., 2020). Specifically, *Ddx3x* silencing *in utero* (Lennox et al., 2020) or forebrain-conditional deficiency (Hoye et al., 2022) result in an excess of cortical neural progenitors at the expense of differentiated neurons. Consistently, cortices from mice with global (Boitnott et al., 2021) or forebrain-conditional (Hoye et al., 2022) ablation of *Ddx3x* are thinner. Considering the role of progenitors in shaping the fate of their post-mitotic progeny (Seuntjens et al., 2009; Telley et al., 2019), it is possible that the alteration in the pool of neurons co-expressing CTIP2 and BRN1 caused by *Ddx3x* mutations depend upon earlier perturbations in neurogenesis. Subsequent alterations ensuing post-mitotically might also contribute, given that *Ddx3x* is also essential for proper neuronal development (Mossa et al., 2024 preprint).

The molecular mechanisms underlying these cellular phenotypes remain unknown. DDX3X is a DEAD-box RNA helicase with critical roles in RNA metabolism, especially mRNA translation (Hoye et al., 2022; Lennox et al., 2020). In addition to well-established transcriptional drivers (Greig et al., 2013; Molyneaux et al., 2015), translational regulation also contributes to shape cortical neuron diversity (Borisova et al., 2024; Duffy et al., 2022; Kraushar et al., 2014; Popovitchenko et al., 2020; Salamon et al., 2023; Zahr et al., 2018). *Ddx3x* modulates the translational efficiency of mRNAs in neural progenitors (Hoye et al., 2022), with a preference for those with highly structured 5′-untranslated regions (Calviello et al., 2021). Intriguingly, a recent study has identified another RNA helicase of the DEAD-box family, eIF4A1, as a factor required for the translation of the *Satb2* mRNA via its structured 5′-untranslated region during cortical development (Borisova et al., 2024). Further, loss of the translational repressor Pumilio2 causes aberrant co-expression of antithetic TFs BRN1 and TLE4 (Zahr et al., 2018). It is plausible that *Ddx3x* might similarly influence the translation of mRNAs dictating neuron molecular identity. Further, mRNA translation rate differs across cortical neurons subtypes, with high rates in layer V CTIP2+ neurons (Borisova et al., 2024). Hence, CTIP2+ neurons might have higher requirements of translational regulators and thus be particularly susceptible to *Ddx3x* perturbations.

To our knowledge, the evidence of altered cortico-brainstem projections (Fig. 4) is the first hint into the circuits that might be altered in *Ddx3x* mutant brains. Given the ubiquitous expression of DDX3X and the evidence of pontine hypoplasia (Lennox et al., 2020; Scala et al., 2019) and/or cerebellar malformations (Aldinger et al., 2019; Lennox et al., 2020) in individuals with *DDX3X* mutations, *Ddx3x* haploinsufficiency also plausibly impacts other elements of the cortico-ponto-cerebellar circuit. While these new observations suggest that the motor phenotypes might have origins *in utero*, there might be still an opportunity to rectify behavioral phenotypes with targeted manipulations of these circuits later in life. Encouraging evidence for potential benefits of intervention on motor circuits in adulthood comes also from our prior work (Boitnott et al., 2021), where we observed a prevention of motor decline in 1-year-old *Ddx3x*^+/−^ mice pre-exposed to behavioral training (Boitnott et al., 2021). This might be particularly relevant for translational approaches, as motor outcomes are objective, measurable in rodents also during postnatal development, and well conserved across species (Dierdorff et al., 2024; Petkova et al., 2022; Thurm et al., 2020). The changes in cortico-brainstem neurons likely have developmental origins. It is reasonable to conjecture that re-expression of DDX3X in postnatal mutant brains would have little effect on the balance of cortical populations. However, *Ddx3x* is also required to maintain the appropriate balance of dendritic spines (Mossa et al., 2024 preprint), therefore re-introducing DDX3X later in life might still ameliorate neuronal connectivity. Attempting the manipulations of these circuits might open new avenues for the development of targeted interventions for DDX3X syndrome.

## METHODS AND MATERIALS

### Mice

All animal procedures were approved by the Institutional Animal Care and Use Committee of the Icahn School of Medicine at Mount Sinai. The *Ddx3x*^flox^ mouse line was generated as previously described (Boitnott et al., 2021). To generate the *Ddx3x* knockout (KO, ­­–) allele, *Ddx3x*^flox/flox^ females were crossed with B6.Cg-Edil3^Tg(Sox2-Cre)1Amc^/J males (*Sox2*-Cre/+) (Hayashi et al., 2002) (The Jackson Laboratory, stock number #008454). To generate the *Ddx3x*^+/+^ and *Ddx3x*^+/−^ female mice in this study, homozygous *Ddx3x*^flox/flox^ were crossed with heterozygous *Sox2*-Cre/+ males. The colony was maintained in a room on a 12 h:12 h light:dark cycle, with lights on at 7:00 am at a constant temperature of 21-22°C and 55% humidity. Standard rodent chow and potable water were available *ad libitum.* Animals were group housed, with a maximum of five mice per cage. Mice were weaned at P21. The colony was maintained on a C57BL/6J background. Genotyping was performed as previously described (Boitnott et al., 2021). All animals used in this study were females.

### Cell birth-dating

A single dose of 5-bromo-2′-deoxyuridine (50 mg/kg) (BrdU; #B5002, Sigma-Aldrich) was intraperitoneally injected in E13.5 or E16 pregnant dams. The brains of P3 female offspring were collected and processed for immunostainings, as outlined below.

### Immunostainings

Embryonic (E17) and early postnatal (P0, P3) mice were euthanized by decapitation. P14 and 4-month-old mice were anesthetized using isoflurane and perfused with 10 ml of phosphate-buffered saline (PBS) followed by 60 ml of ice-cold 4% paraformaldehyde (PFA). Brains were fixed in 4% PFA overnight at 4°C and then cryopreserved in 30% (w/vol) sucrose, 0.05% sodium azide, and 100 mM glycine in PBS. Brains were embedded in Tissue Plus OCT Compound (#4585, ThermoFisher Scientific) and sectioned coronally using a Leica CM1860 cryostat at 40 μm thickness (E17, P0, P3) or 30 μm thickness (P14, 4-month-old). Sections were washed in PBS with 0.05% Triton X-100 (#X-100, ThermoFisher Scientific) and blocked with 5% donkey serum (#D9663, Sigma-Aldrich) for 1 h at room temperature. Sections were incubated overnight at 4°C with primary antibodies. The next day, after washing in PBS with 0.05% Triton X-100, sections were incubated for 2 h at room temperature with the corresponding secondary antibodies, washed and incubated with Hoechst 33342 (1/2000; #H3570, Invitrogen). Sections were mounted on coverslips with Fluoromount-G (#0100-01, SouthernBiotech). Primary antibodies used were anti-CTIP2/BCL11B (rat monoclonal; 1:300; # 650602, BioLegend), anti-BRN1/POU3F3 (goat polyclonal; 1:100; #NBP1-49872, Novus Biosystems), anti-BrdU (mouse monoclonal; 1:100; #347580, BD Biosciences), and/or anti-SATB2 (mouse monoclonal; 1:400; #ab51502, Abcam). Secondary antibodies used were anti-mouse Alexa Fluor 488 (#A21202, 1:200, Invitrogen), anti-rat Alexa Fluor 647 (#ab150155, 1:200, Abcam) and anti-goat Alexa Fluor 568 (#A11057, 1:200, Invitrogen).

### Retrograde tracing

5-week-old mice were placed in an incubation chamber and anesthetized with a mixture of oxygen and isoflurane (5% induction and 1% maintenance). They were then placed in the stereotaxic apparatus for injections of retrograde AAV-CAG-GFP viral particles (#37825-AAVrg, Addgene) (0.5 μl; rate of 0.1 μl/min) using a 5 μl Hamilton microliter syringe. The needle was left in place for 5 min post-injection to ensure a proper diffusion of the virus into the tissue. The pontine reticular nucleus (PRNr) was targeted using the coordinates AP −4.00, ML 0.75, DV 4.50, based on the Allen Brain Atlas. To ensure successful viral expression, mice were perfused and brains collected for posterior analysis 3 weeks after the stereotaxic injection.

### Confocal imaging and processing

Images of whole cortices were acquired using a Leica TCS SP8 laser scanning confocal microscope (Leica Microsystems Heidelberg GmbH, Manheim, Germany) coupled to a Leica DMi8 inverted microscope at 10× and 63× magnification. Argon, 561 nm and 633 nm lasers and HyD detectors were used. Images were acquired at 600 Hz speed and 1024×1024 pixels. GIMP software was used for binning medial/primary motor cortex (MOp) and lateral/primary somatosensory cortex (SSp). Media/MOp and lateral/SSp were identified using the Allen Mouse Brain atlas. To normalize all bins across all postnatal samples and sections, the beginning of the M1 bin started 100 μm from the peak of the cingulum bundle and spanned 340 μm wide. The bin for SSp started 100 μm from the lateral end of the cingulum bundle and spanned 340 μm wide (P3, P14, and 4-month-old; Figs 2 and 3) or 850 μm wide (Fig. 4). For embryonic sections, the bin for medial cortex started at the most upper and medial part of the cortex and spanned 230 μm wide, and the bin for lateral cortex started at the limit between the ganglionic eminence and the cortex and spanned 320 μm wide. Each region was binned evenly into 10 bins. Quantifications were conducted on 6-8 sections/animal and averaged across sections. All images were analyzed using the Cell Counter plugin in ImageJ software version 1.51 (Schindelin et al., 2012).

### Statistical analyses

All statistics and plots were generated using custom R scripts and the ggplot package. Statistical tests, number of animals in each experiment, and significance are indicated in each figure and/or corresponding legend. Data are shown as mean±s.e.m. Outliers are defined as data points below Q1-1.5×IQR or above Q3+1.5×IQR, where Q1 is the first quartile, Q3 is the third quartile, and the IQR is the interquartile range. Outliers, shown in plots as grey data points, were removed from the calculations of the mean and s.e.m. and for the statistical tests. Shapiro–Wilk test was used to assess normality and Bartlett's test to assess homoscedasticity. In case of multiple comparisons, Kruskal–Wallis test was employed for non-parametric data, Welch ANOVA for normally distributed data with unequal variance, and ANOVA test for normally distributed data with equal variance, followed by Tukey's honest significant difference test. Groups comparisons were then conducted with Student's *t*-test for normally distributed data with equal variance, Welch's two sample *t*-test for normally distributed data with unequal variance, or Wilcoxon signed-rank test for not normally distributed data, with Benjamini-Hochberg correction for multiple comparisons. Whenever possible, data are reported by individual (not only by group).

## Supplementary Material

10.1242/biolopen.061854_sup1 Supplementary information
