# Supplementary material for: A subpopulation of cortical neurons altered by mutations in the autism risk gene DDX3X
Source: Biol Open. 2025 Jan 29;14(1):bio061854. doi: 10.1242/bio.061854 (PMC11815569; doi:10.1242/bio.061854)
Supplement: Supplementary information [file biolopen-14-061854-s1.pdf]

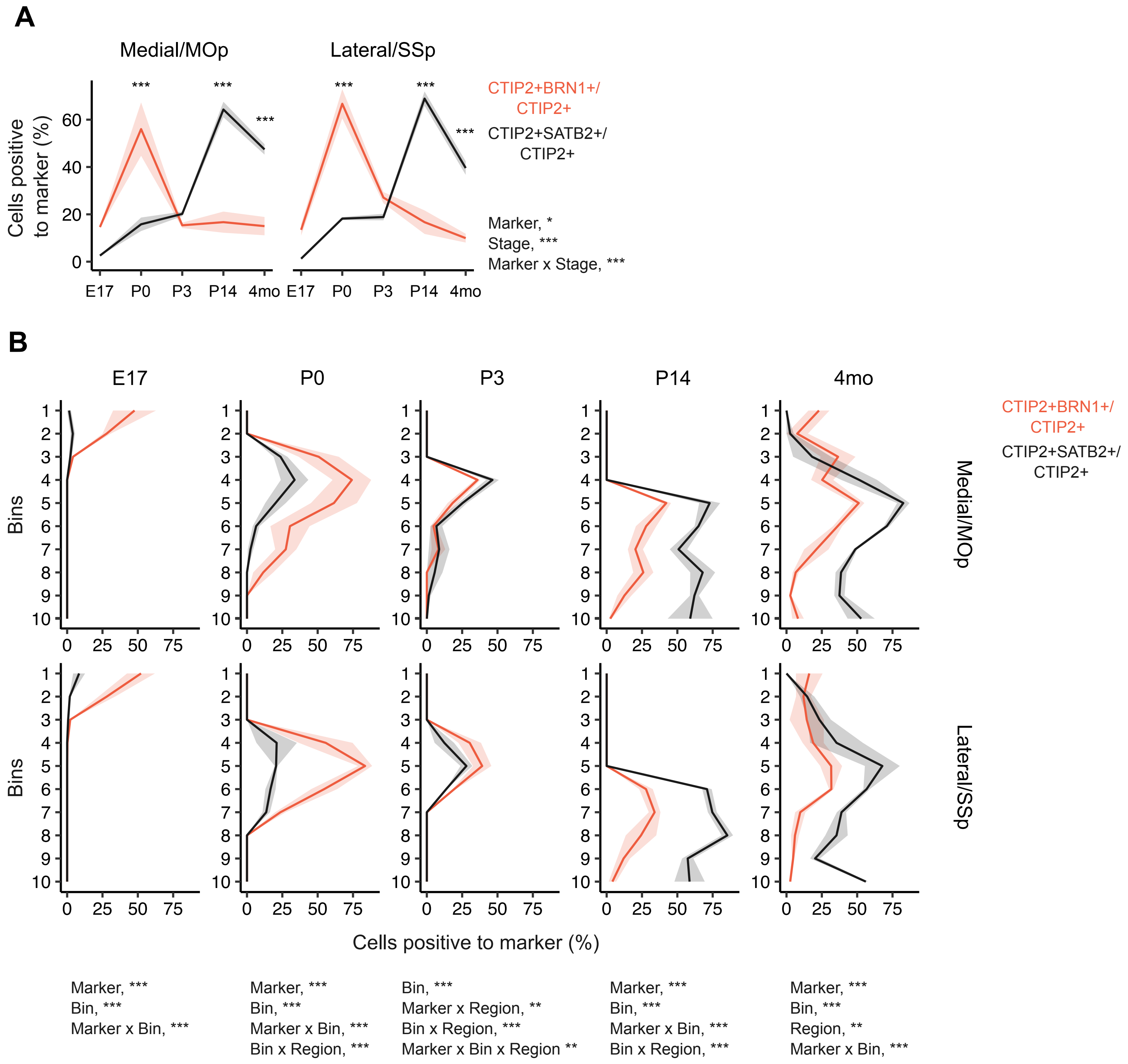

Fig. S1. Subpopulations of layer V neurons change dynamically during cortical development. A) Percentage of CTIP2+BRN1+ (red) and CTIP2+SATB2+ (grey) over all CTIP2+ cells at the the developmental stages indicated in Figure 1 and in both medial/MOp (left panel) and lateral/SSp (right panel) regions of the cortex. *n*=3 mice/experiment; mean ± SEM; ANOVA for marker (*P*-value<0.05), region (not significant), developmental stage (*P*-value<0.001), and their interactions (only marker x stage significant, *P*-value<0.001), followed by Tukey's 'Honest Significant Difference' test for marker within each developmental stage. **P*-value<0.05; ****P*-value<0.001. **B**) Laminar distribution of the percentage of CTIP2+BRN1+ (red), and CTIP2+SATB2+ (grey) over all CTIP2+ cells at the developmental stages indicated in Figure 1 and in both medial/MOp (upper panel) and lateral/SSp (lower panel) regions of the cortex, from the pia (Bin 1) to the ventricle (Bin 10). *n*=3 mice/experiment; mean ± SEM; Multi-way ANOVA for marker, region, bins, developmental stage, and their interactions (only significant variables shown below the plots); **P*-value<0.05, ***P*-value<0.001, ****P*-value<0.001.

Biology Open • Supplementary information
